# Increasing Prevalence of Potential Vitamin D Toxicity and Its Risk Factors in Korea: A Large Population-Based Study

**DOI:** 10.3390/nu17162614

**Published:** 2025-08-12

**Authors:** Rihwa Choi, Gayoung Chun, Sung-Eun Cho, Sunhyun Ahn, Sang Gon Lee, Eun Hee Lee

**Affiliations:** 1 Laboratory Medicine Center, Division of Laboratory Medicine, GC Labs, Yongin-si 16924, Republic of Korea; pirate0720@naver.com (R.C.); ash1008@gclabs.co.kr (S.A.); ehlee@gclabs.co.kr (E.H.L.); 2 Department of Laboratory Medicine and Genetics, Sungkyunkwan University School of Medicine, Seoul 06351, Republic of Korea; 3 Biostatistics Team, Infectious Disease Research Center, Division of Laboratory Medicine, GC Labs, Yongin-si 16924, Republic of Korea; forjund@gclabs.co.kr; 4 Endocrine Substance Analysis Center (ESAC), Division of Laboratory Medicine, GC Labs, Yongin-si 16924, Republic of Korea

**Keywords:** 25-hydroxyvitamin D, supplementation, toxicity, nutritional epidemiology

## Abstract

**Background/Objectives**: Vitamin D plays an important role in a wide range of health outcomes, including immune regulation, bone metabolism, cardiovascular health, and cancer prevention. However, recent data on the prevalence and risk factors for elevated serum 25-hydroxyvitamin D [25(OH)D] concentrations—indicative of potential vitamin D toxicity—remain limited in Korea. **Methods**: We conducted a retrospective analysis of laboratory data from individuals who underwent serum 25(OH)D testing between 2020 and 2022. Potential vitamin D toxicity was defined as serum 25(OH)D levels exceeding 50, 100, or 150 ng/mL. The prevalence of potential toxicity was examined by age, sex, test month, and year. **Results**: A total of 1,198,947 individuals (mean age, 45.7 ± 19.4 years; 31.6% male) were included in the study. The prevalence of serum 25(OH)D > 50 ng/mL increased from 4.41% in 2020 to 6.21% in 2022, and that of >100 ng/mL rose from 0.09 to 0.12% over the same period. The proportions exceeding 50 and 100 ng/mL also rose significantly (*p* < 0.05), while >150 ng/mL remained rare (0.01%) and stable. Elevated 25(OH)D concentrations were more frequently observed among females, children aged 0–9 years, and adults aged ≥50 years (adjusted odds ratios for multivariable logistic regression > 1.0, *p* < 0.05). **Conclusions**: Although the prevalence of potential vitamin D toxicity remains low, its continuous upward trend highlights the need for increased public awareness, clinical monitoring, and guideline-based supplementation strategies to prevent inadvertent vitamin D intoxication, particularly in the aging population.

## 1. Introduction

Vitamin D has garnered global attention due to its association with a wide range of health outcomes, including immune regulation, bone metabolism, cardiovascular health, and cancer prevention [1,2,3]. Numerous studies have explored its role in both disease prevention and therapeutic modalities across diverse populations worldwide [1,2,3,4,5]. Vitamin D deficiency has been linked to increased risk of osteoporosis, rickets, autoimmune diseases, infectious diseases such as tuberculosis and COVID-19, as well as metabolic conditions including diabetes and obesity [1,2,3,4,5,6,7]. In therapeutic contexts, vitamin D supplementation has been investigated for its potential to reduce fracture risk in the elderly, modulate immune responses in patients with multiple sclerosis or inflammatory bowel disease, and improve insulin sensitivity in individuals with metabolic syndrome [1,2,3,4,5,6,7].

Exposure to ultraviolet B light triggers vitamin D production from 7-dehydrocholesterol in the skin, followed by hepatic and renal hydroxylation to generate its active form [6,7]. In the liver, cholecalciferol is hydroxylated by 25-hydroxylase (CYP2R1) to form 25-hydroxyvitamin D [25(OH)D], which is further hydroxylated in the kidneys by 1α-hydroxylase (CYP27B1) to produce the active hormone, 1,25-dihydroxyvitamin D [1,25(OH)_2_D] [6,7]. This active form binds to the vitamin D receptor (VDR), regulating the expression of target genes involved in calcium homeostasis and immune function [6,7]. Genetic variations in vitamin D metabolism genes (e.g., CYP2R1, CYP27B1, CYP24A1) and VDR may influence individual responses to vitamin D and susceptibility to deficiency or toxicity [6,7,8].

Although multiple forms of vitamin D exist in the body, 25(OH)D is used to assess vitamin D status because it is the major circulating form, reflects total vitamin D input from both endogenous synthesis and dietary sources, and has a relatively long half-life [6,7,8,9]. However, due to substantial inter- and intra-individual variability in vitamin D metabolism driven by genetic, physiological, and environmental factors, the optimal serum 25(OH)D concentration that defines sufficiency remains a subject of ongoing debate across countries and clinical guidelines [6,7,8,9,10]. The U.S. National Health and Nutrition Examination Survey (NHANES), conducted by the Centers for Disease Control and Prevention (CDC), considers serum 25(OH)D levels between 20 and 50 ng/mL to indicate vitamin D sufficiency, while the Korea National Health and Nutrition Examination Survey (KNHANES) considers levels between 30 and 50 ng/mL to indicate sufficiency [11,12,13].

Most previous studies have focused on vitamin D deficiency and its association with various health outcomes [1,2,3,4,5]. In recent decades, there has been a growing interest in and increased prescription of vitamin D globally [2,3,14,15]. Vitamin D toxicity is rarely attributed to cutaneous synthesis or dietary intake alone; instead, it is predominantly associated with excessive exposure to pharmacologic doses of vitamin D supplements [6,8,9,15]. Recently, the growing use of vitamin supplements among older adults has raised concerns about the potential for misuse and inadvertent toxicity [14,15,16].

Meanwhile, there is no globally standardized threshold for vitamin D toxicity, as significant inter- and intra-individual variability may result in some individuals exhibiting no clinical signs or symptoms even at markedly elevated serum 25(OH)D levels [6,8,9,10]. A retrospective population-based study conducted in the United States between 2002 and 2011 reported a significant increase in the incidence of serum 25(OH)D levels exceeding 50 ng/mL, without a corresponding rise in cases of acute clinical toxicity [14].

Although vitamin D is essential for maintaining calcium homeostasis and skeletal health, excessive intake can lead to toxicity, particularly when obtained through high-dose supplementation [6,8,9,16,17]. Vitamin D toxicity is primarily characterized by hypercalcemia, which may result in nausea, vomiting, polyuria, dehydration, and muscle weakness [6,8]. In more severe cases, it can progress to nephrocalcinosis, acute kidney injury, cardiac arrhythmias, and neuropsychiatric symptoms such as confusion or even coma [6,8,9,15,16,17]. These adverse effects are not typically observed with endogenous synthesis from sunlight or dietary intake alone, but rather from prolonged ingestion of pharmacologic doses of vitamin D supplements [6,8,9,15,16,17]. The threshold for toxicity varies across individuals, depending on factors such as age, renal function, genetic variations in vitamin D metabolism, and concurrent use of medications that affect calcium or vitamin D homeostasis [6].

While rare, fatal cases of vitamin D intoxication have been reported, underscoring the need for appropriate monitoring, especially in populations with increased supplementation use [15,16,17]. Given the possibility of fatal complications, it is important to exercise clinical vigilance and implement careful monitoring when serum 25(OH)D concentrations exceed the lowest levels associated with toxic effects [15]. Serum levels exceeding 100 ng/mL are commonly used as a threshold for potential toxicity in Europe and Korea [9,13,14,16]. In the U.S. NHANES program, serum 25(OH)D levels exceeding 50 ng/mL are regarded as potentially concerning, as emerging evidence links such high levels to adverse health effects [11].

As Korean society undergoes rapid aging, public awareness of and medical interest in bone health—especially osteoporosis prevention—have significantly increased [11,12,13]. Consequently, the consumption of vitamin D supplements and other health-functional foods has risen in recent years [12,18]. While numerous Korean studies have investigated the prevalence and health implications of vitamin D deficiency, recent data on vitamin D toxicity remain scarce, and information on its epidemiological burden and risk factors is limited. Therefore, this study aimed to estimate the recent prevalence of potential vitamin D toxicity in the Korean population and to identify associated risk factors contributing to elevated serum 25(OH)D concentrations, using various cutoff levels for the assessment of possible toxicity.

## 2. Materials and Methods

This retrospective study analyzed serum 25(OH)D test results from Korean individuals referred by local clinics and hospitals to GC Labs between January 2020 and December 2022. Samples with missing age or sex information were excluded from the analysis. All data were fully anonymized prior to analysis in accordance with ethical standards for secondary use of clinical laboratory data. We analyzed the initial 25(OH)D measurements for individuals with multiple tests to estimate the prevalence of possible vitamin D toxicity.

All assays were performed using the same analytical platform throughout the three-year study period, utilizing the Architect i2000SR system (Abbott Diagnostics, Abbott Park, IL, USA) [5]. The analytical accuracy of serum 25(OH)D measurements was periodically evaluated through method comparison studies against liquid chromatography–tandem mass spectrometry (LC-MS/MS) and was further assured through participation in the accuracy-based external quality assessment program, the Vitamin D Standardization Certification Program (VDSCP) administered by the U.S. CDC [19].

To assess possible vitamin D toxicity, serum 25(OH)D concentrations were classified based on commonly referenced thresholds in international guidelines and literature. The following cutoffs were applied: >50 ng/mL (125 nmol/L), often considered above the range of sufficiency; >100 ng/mL (250 nmol/L), associated with increased risk of toxicity; and >150 ng/mL (375 nmol/L), generally regarded as a potentially toxic level [6,9,12,15,16]. For international comparison, values in ng/mL were converted to nmol/L using the standard conversion factor (1 ng/mL = 2.5 nmol/L).

Descriptive statistics were used to estimate the prevalence of samples exceeding each vitamin D cutoff, stratified by age, sex, month of testing, and year. Age was categorized into nine groups in 10-year increments: 0–9, 10–19, 20–29, 30–39, 40–49, 50–59, 60–69, 70–79, and ≥80 years.

Multivariable logistic regression analysis was conducted to identify factors independently associated with serum 25(OH)D levels exceeding each threshold. Results were reported as odds ratios (ORs) with 95% confidence intervals (CIs). A *p*-value of <0.05 was considered statistically significant. All statistical analyses were performed using R software (version 4.5.1; R Foundation for Statistical Computing, Vienna, Austria).

## 3. Results

During the three-year study period (2020–2022), a total of 1,572,157 serum (OH)D measurements were performed on 1,198,947 individuals. Among the total 1,198,947 individuals, 942,083 had only one 25(OH)D measurement during the 3-year study period, while the remaining 256,864 had two or more measurements (median number of measurements: 2; interquartile range [IQR]: 2–2), resulting in a total of 630,074 test records from this group. The mean age of the tested population was 45.7 years (standard deviation [SD], 19.4), comprising 378,304 males (31.6%, mean age 45.0 years [SD 20.5]) and 820,643 females (68.4%, mean age 46.0 years [SD 18.8]). The overall mean serum 25(OH)D concentration was 25.4 ± 13.7 ng/mL. Table 1 summarizes the baseline demographic characteristics of the study population.

**Figure 1:**
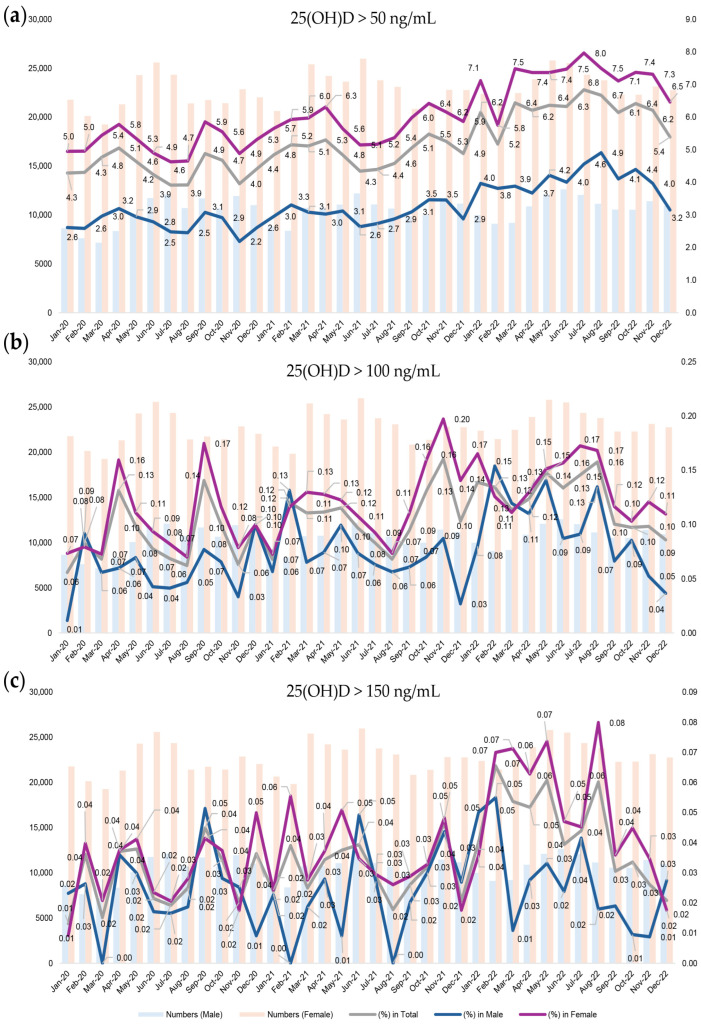
Monthly and sex-specific prevalence of elevated serum 25-hydroxyvitamin D [25(OH)D] levels based on toxicity cutoffs (**a**) >50 ng/mL, (**b**) >100 ng/mL, and (**c**) >150 ng/mL.

Using the first available 25(OH)D measurement per individual, the monthly and sex-specific distributions of serum 25(OH)D concentrations were analyzed. The prevalence of individuals exceeding each cutoff for possible vitamin D toxicity (>50, >100, and >150 ng/mL) is presented in Figure 1. Over the three-year study period, the prevalence varied by month (reflecting seasonal patterns) and showed a gradually increasing trend. While the proportions of individuals exceeding >50 and >100 ng/mL steadily increased over time, levels > 150 ng/mL were extremely rare throughout the study period. Throughout most of the study period, the proportion of individuals with high 25(OH)D levels was higher in females than in males. Due to the low overall frequency of values > 150 ng/mL, there were some months in which no cases were observed among males.

**Figure 2:**
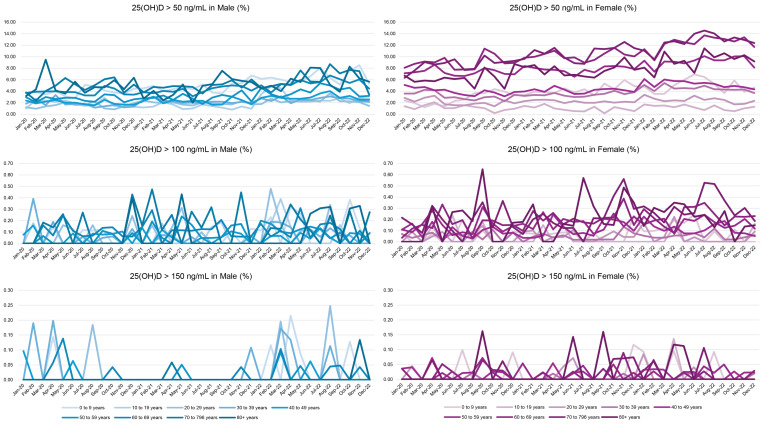
Prevalence of elevated serum 25-hydroxyvitamin D [25(OH)D] levels by age group, sex, and month of testing.

The prevalence of elevated 25(OH)D levels based on each toxicity cutoff (>50, >100, and >150 ng/mL) by sex, month of testing, and age group is presented in Figure 2. Darker solid lines indicate older age groups, among which the proportion of individuals with high 25(OH)D levels was generally higher. These groups also showed a gradual increase in prevalence over recent years. Due to the very low frequency of values > 150 ng/mL, some age groups or test months had no observed cases, resulting in fluctuations in the proportion over time.

**Figure 3:**
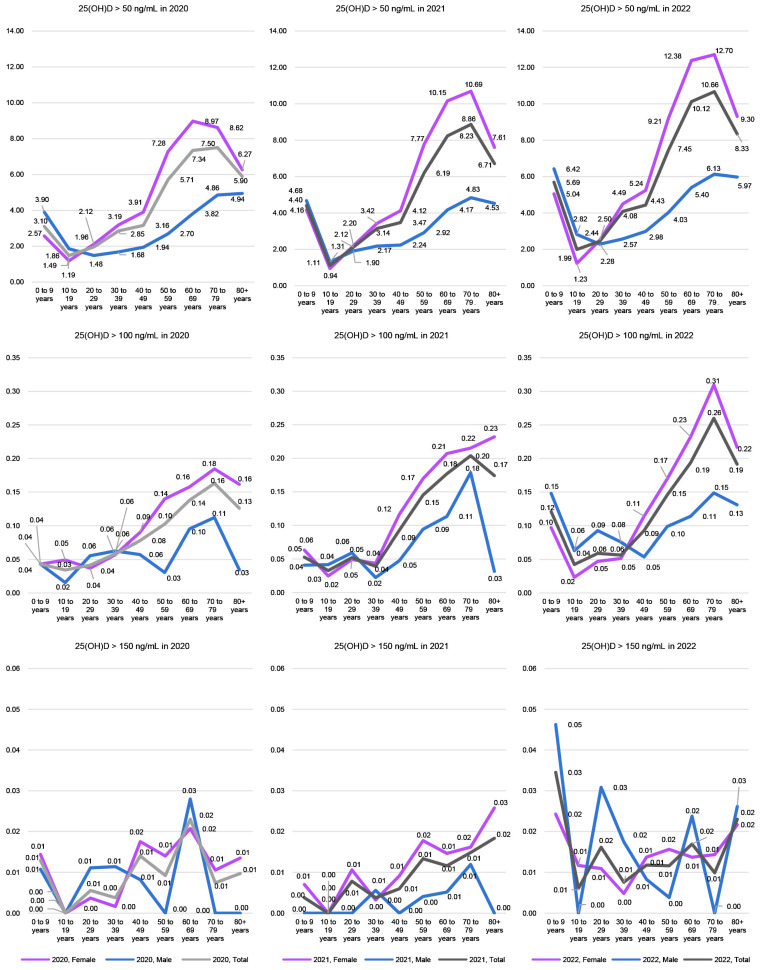
Annual trends in the prevalence of elevated serum 25-hydroxyvitamin D [25(OH)D] levels by age group and sex from 2020 to 2022.

Figure 3 illustrates the annual trends in the proportion of individuals exceeding each 25(OH)D cutoff across age groups. The proportion of individuals with serum 25(OH)D levels > 50 ng/mL was higher in 2022 than in 2020 across all age and sex groups. A similar trend was observed for levels > 100 ng/mL, although in some age groups, the proportion decreased or peaked in 2021. For levels > 150 ng/mL, the overall frequency was very low, leading to greater variability across male age groups. Nonetheless, the general age-related pattern of elevated 25(OH)D—slightly higher in the 0–9-year group, lower in adolescents (10–19 years), and increasing from age 40–49 onward—was consistently observed among female subjects.

**Figure 4:**
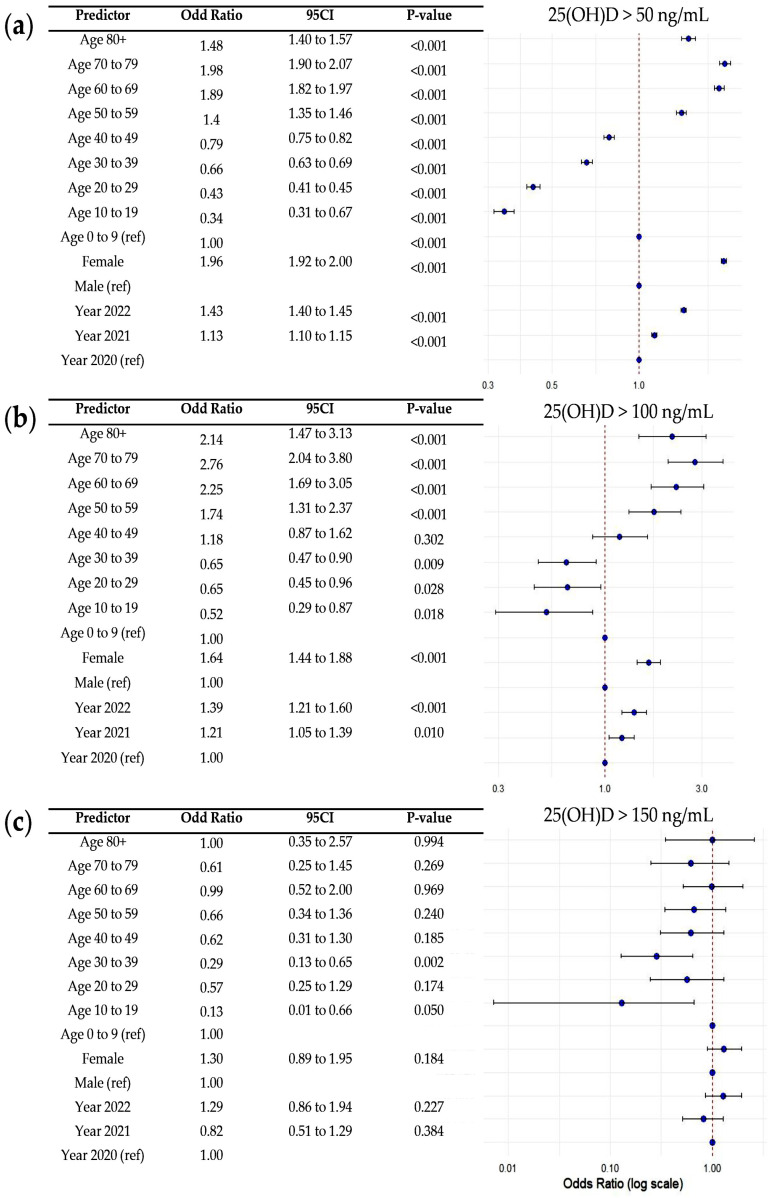
Forest plots of adjusted odds ratios (ORs) and and 95% confidence intervals for factors associated with elevated serum 25-hydroxyvitamin D [25(OH)D] levels, based on multivariable logistic regression analysis: (**a**) >50 ng/mL, (**b**) >100 ng/mL, and (**c**) >150 ng/mL. Reference categories are indicated on the plot. In the plot, blue dots represent the adjusted odds ratios (ORs), dashed vertical lines indicate the reference line at OR = 1.00, and solid horizontal lines represent the 95% confidence intervals for each OR.

To determine factors independently associated with elevated serum 25(OH)D concentrations (>50, >100, and >150 ng/mL), multivariable logistic regression analysis was conducted. As shown in Figure 4, older age groups were significantly associated with higher odds of elevated 25(OH)D levels, particularly among individuals aged 50 years and above. Female sex was also associated with increased odds compared to males (adjusted OR > 1.0 for >50 and >100 ng/mL; *p* < 0.05). In addition, the odds of elevated 25(OH)D progressively increased from 2020 to 2022, reflecting a temporal trend in vitamin D status (adjusted OR > 1.0 for >50 and >100 ng/mL; *p* < 0.05).

## 4. Discussion

In this large-scale retrospective study of over 1.1 million individuals tested over a three-year period, we observed a steady annual increase in the proportion of individuals exceeding both 50 and 100 ng/mL of serum 25(OH)D, across all sexes. Although the proportion of individuals exceeding 150 ng/mL remained very low throughout the study period, this level was consistently detected each year, suggesting that while rare, potential vitamin D toxicity does occur in the general population. These findings are consistent with previous international reports that have raised concerns about the increasing prevalence of high serum 25(OH)D levels in parallel with the widespread use of vitamin D supplements, particularly in older adults and children [10,15,16]. Previous studies on vitamin D toxicity are mostly limited to case reports or small case series [6,8,17,20]. Among the few studies that reported patients with elevated serum 25(OH)D concentrations, the applied cutoffs and reported prevalence rates varied considerably across studies [6,8,15,16,17,20]. In a retrospective study conducted at a university hospital in the United States, 127,932 test results from 73,779 patients were analyzed. The prevalence of serum 25(OH)D > 80 ng/mL was 1.05% and of >120 ng/mL was 0.12%, which is comparable to the prevalence of >100 ng/mL (0.12%) reported in our study [21]. An analysis of the Swedish National Patient Register between May 2010 and July 2011, which included 2003 individuals and measured serum 25(OH)D levels using LC-MS/MS in 268 participants, reported a maximum concentration of 168 nmol/L (67.2 ng/mL), with no cases exceeding 100 ng/mL [22]. According to the publicly available UK Biobank dataset (June 2018 release), serum vitamin D concentrations were reported for 465,069 samples from 449,640 participants, measured using the LIAISON XL immunoassay (DiaSorin S.p.A., Saluggia, Italy). The 90th percentile (Decile 9) value was 76.6 nmol/L (30.64 ng/mL), and only 610 samples (0.13%) exceeded 130.8 nmol/L (52.32 ng/mL), indicating a substantially lower prevalence of elevated 25(OH)D levels compared to our study findings [23].

In the present study, the proportion of individuals with serum 25(OH)D concentrations > 50 and >100 ng/mL increased over the study years, a trend consistent with findings from the U.S. NHANES [12,15,16,24]. NHANES data from adults aged ≥18 years between 2007 and 2018 similarly demonstrated increases in both vitamin D intake and serum 25(OH)D concentrations over time [12,24].

Supporting our findings, data from the Health Insurance Review and Assessment Service (HIRA) healthcare big data open system in Korea indicate a striking increase in the number of patients diagnosed with hypervitaminosis D (International Classification of Diseases, 10th Revision [ICD-10] code E67.3) over the past decade [20]. Specifically, the number of individuals receiving medical care for vitamin D toxicity rose from only 3 cases in 2010 to 106 in 2024, with the highest number—135 cases—recorded in 2022 [20]. Notably, the majority of these cases occurred in females and in two distinct age groups: young children aged 0–9 years and adults aged ≥50 years. This is consistent with the high-risk subgroups identified in our study. Several previously published case series have reported on hypervitaminosis D, including a study from India in which 16 individuals with serum 25(OH)D levels > 150 ng/mL were identified using the Abbott Architect i1000 SR platform between January 2011 and January 2013 [25]. The median age of these individuals was 64.5 years (range: 42–86 years), and the sex distribution was relatively balanced, with seven females (43.8%) [25]. In another hospital-based study conducted between November 2022 and October 2024, 1384 children were evaluated using the Vitros 5600 platform (Ortho Clinical Diagnostics, Mexico) [26]. The investigators reported that younger children exhibited higher median serum 25(OH)D concentrations, which is consistent with our findings. In that study, hypervitaminosis D, defined as serum 25(OH)D > 100 ng/mL, was observed in 1.5% of participants—a higher prevalence than in our cohort—but the trend of increased frequency in younger age groups was similar to that observed in our study [26].

These real-world clinical data reinforce the need for further research and the development of practical guidelines regarding serum 25(OH)D monitoring in these vulnerable populations. The latest 2024 Endocrine Society Clinical Practice Guideline recommends against vitamin D testing and supplementation beyond dietary reference intake in healthy adults under the age of 75, advising instead a targeted approach for specific risk groups while discouraging routine serum 25(OH)D screening in the general healthy population [1,27,28]. However, in populations with specific medical conditions, follow-up monitoring of serum 25(OH)D levels is recommended [29,30]. In Korea, serum 25(OH)D testing is reimbursed in patients with conditions affecting vitamin D metabolism or absorption, such as gastrointestinal disorders, chronic liver or kidney disease, malignancy, metabolic bone disorders, and in those receiving medications known to interfere with vitamin D pathways [31]. For patients undergoing vitamin D supplementation, insurance coverage allows testing once before treatment, once at 3–6 months to assess response, and up to twice annually for long-term monitoring [31]. We identified subpopulations in which elevated 25(OH)D levels were more frequently observed—namely, females, young children aged 0–9 years, and older adults aged 50 years and above. These groups may require greater clinical attention in terms of vitamin D monitoring, particularly in the context of prolonged or high-dose supplementation. The present study provides baseline epidemiological data that may serve as a useful reference for clinicians and public health professionals in evaluating risk and establishing appropriate monitoring strategies for individuals receiving vitamin D supplements.

Although the actual levels of vitamin D supplementation or intake in the study population were not available, the Korean Nutrition Society provides Korean Dietary Reference Intakes (KDRIs) and develops educational programs and materials targeted at the general public as well as high-risk groups, including children, adolescents, and patients with cancer [32]. The KDRIs are revised every five years, and the most recent versions currently available are the 2020 KDRIs [32]. The next revision, scheduled for 2025, is currently in progress [32]. Globally, various intake guidelines for vitamin D are applied, and the KDRIs recommend an adequate intake ranging from 5 to 15 µg/day (200–600 IU/day) and a tolerable upper intake level ranging from 25 to 100 µg/day (1000–4000 IU/day), depending on age, sex, and pregnancy status [1,2,3,4,5,6,7,10,11,32]. These values are generally consistent with internationally recommended levels [1,2,3,4,5,6,7,10,11,32]. Based on the 2020 KNHANES 24 h dietary recall data from 5808 participants, the primary dietary sources of vitamin D in Korea were seafood (58.6%), eggs (20.5%), and meat (9.4%), which is consistent with previously reported major sources of vitamin D intake globally and in Japan [6,33,34]. The report noted that older adults aged ≥65 years consumed less than 20% of the recommended adequate intake, indicating a significant deficiency in this age group [33]. When considered alongside the findings of the present study, which identified a higher prevalence of elevated serum 25(OH)D levels among older adults, this suggests that both deficiency and excessive levels may coexist in the elderly population, underscoring the importance of appropriate monitoring in this age group [6,17,25]. The Korean Academy of Medical Sciences, in its Evidence-Based Recommendations for Osteoporosis in Primary Care, recommends calcium and vitamin D supplementation for individuals with osteopenia or osteoporosis [35]. For fracture prevention in high-risk groups—including men aged ≥50 years and postmenopausal women—a target serum 25(OH)D concentration of 30–50 ng/mL is recommended, with individualized blood testing and vitamin D dosing [35]. Although there is currently no globally unified guideline, this range is consistent with the concentrations suggested by the U.S. NHANES [12,27].

This study has several limitations. First, the data were derived from a clinical laboratory database, which may include individuals tested for specific clinical indications rather than a random sample of the general population. The study population consisted primarily of individuals whose specimens were submitted from local clinics and hospitals and may not fully represent patients in tertiary care or academic medical centers, such as university hospitals. The present study includes a substantially higher proportion of female participants compared to males, which may reflect health-seeking behaviors or vitamin D testing patterns in real-world clinical settings but may also limit the generalizability of the findings. As this study was a retrospective analysis based solely on laboratory data, we were unable to ascertain whether individuals had specific underlying conditions such as osteoporosis, chronic kidney disease, or other disorders that may affect vitamin D metabolism or testing frequency. This limitation hinders a comprehensive interpretation of the clinical context of elevated serum 25(OH)D concentrations and should be taken into account when generalizing the findings. Additionally, the extremely low frequency of serum 25(OH)D concentrations exceeding 150 ng/mL limited the ability to perform robust subgroup analyses, particularly among males. In some months or subgroups, no cases were observed, which may have impacted the statistical power and the reliability of temporal or sex-specific comparisons at this cutoff level. Information on vitamin D supplementation dose, duration, dietary intake, sun exposure, comorbidities, and clinical symptoms was not available, limiting causal interpretation [6,27]. As clinical data on supplement intake were not available in the laboratory database, we were unable to assess the relationship between supplement use and elevated serum 25(OH)D concentrations. This limitation hinders our ability to determine whether high levels were due to exogenous supplementation, underlying clinical conditions, or other factors [6,27]. In addition, information on dietary habits—which can significantly influence serum 25(OH)D levels through intake of vitamin D–rich foods or fortified products—was not available in the present dataset. As a result, we were unable to account for the potential contribution of diet to vitamin D status, which represents another limitation of this study. Therefore, caution should be exercised in extrapolating these results to all clinical settings.

Despite these limitations, the large sample size strengthens the value of our findings. Future research should incorporate clinical outcomes, detailed supplement histories, and genomic or metabolic profiles to better define the risk of vitamin D toxicity [6]. In addition, longitudinal studies are warranted to establish optimal monitoring intervals and thresholds for safe supplementation in specific at-risk populations, including young children, older adults, and females [6,16,17].

## 5. Conclusions

This large-scale, laboratory-based study demonstrated a gradual and consistent increase in the prevalence of elevated serum 25(OH)D levels in the Korean population from 2020 to 2022, particularly above the cutoffs of 50 and 100 ng/mL. Although levels exceeding 150 ng/mL remained rare, such values were detected each year and warrant continued attention. Elevated 25(OH)D concentrations were more frequently observed among females, children aged 0–9 years, and adults aged ≥50 years. These findings highlight the need for more targeted monitoring strategies and evidence-based guidelines for safe vitamin D use in these vulnerable groups. Further longitudinal and outcome-based studies are essential to define optimal monitoring intervals and to better understand the clinical consequences of excess vitamin D exposure in the general population.

## Figures and Tables

**Table 1:** Baseline characteristics of the study population (2020–2022, total *n* = 1,198,947).

Category	Characteristics	*n*	(%)
Year	2020	387,850	32.3
	2021	400,478	33.4
	2022	410,619	34.2
Sex	Male	378,304	31.6
	Female	820,643	68.4
Age group	0 to 9 years	72,934	6.1
	10 to 19 years	46,385	3.9
	20 to 29 years	111,892	9.3
	30 to 39 years	238,485	19.9
	40 to 49 years	199,593	16.6
	50 to 59 years	228,991	19.1
	60 to 69 years	181,983	15.2
	70 to 79 years	84,392	7.0
	80+ years	34,292	2.9
25(OH)D	>50 ng/mL	62,275	5.19
	>100 ng/mL	1269	0.12
	>150 ng/mL	129	0.01

## Data Availability

The datasets generated and analyzed during the current study are available from the corresponding authors on reasonable request. The data are not publicly available due to privacy or ethical restrictions.

## Abbreviations

The following abbreviations are used in this manuscript:

25(OH)D	25-hydroxyvitamin D
CDC	Centers for Disease Control and Prevention
KDCA	Korea Disease Control and Prevention Agency
KNHANES	Korea National Health and Nutrition Examination Survey
NHANES	National Health and Nutrition Examination Survey
